# Leveraging reimbursement strategies to guide value-based adoption and utilization of medical AI

**DOI:** 10.1038/s41746-022-00662-1

**Published:** 2022-08-10

**Authors:** Kaushik P. Venkatesh, Marium M. Raza, James A. Diao, Joseph C. Kvedar

**Affiliations:** Harvard Medical School, Boston, MA USA

**Keywords:** Health policy, Health care economics

## Abstract

With the increasing number of FDA-approved artificial intelligence (AI) systems, the financing of these technologies has become a primary gatekeeper to mass clinical adoption. Reimbursement models adapted for current payment schemes, including per-use rates, are feasible for early AI products. Alternative and complementary models may offer future payment options for value-based AI. A successful reimbursement strategy will align interests across stakeholders to guide value-based and cost-effective improvements to care.

Artificial intelligence (AI) tools for health care are defined as technologies that learn from data to produce clinical predictions and recommendations that mimic human cognition. These AI systems have grown exponentially over the past decade, with 130 AI system “devices” approved by the US Food and Drug Administration (FDA) between 2015 and 2020^1^. With the increasing availability of AI awaiting integration into clinical practice, financing these new technologies has become a chief concern. In 2020, the Centers for Medicare and Medicaid Services (CMS) agreed to cover the first Common Procedural Terminology code and New Technology Add-On Payment (NTAP) for an AI system^2^. These provide per-use reimbursement for AI systems and expected costs above Diagnosis-Related Group (DRG) amounts, respectively. Although these recent precedents may shed some light on CMS’s reimbursement decision framework, the agency has not yet issued guidance on how it will continue to evaluate submissions for reimbursement.

Payment strategies have important consequences for utilization, cost, and quality of care across the wide range of clinical use cases for AI. Future payment models should account for AI’s unique properties, including its near-zero marginal cost, to discourage overuse^3^. Two recent papers in *npj Digital Medicine* stimulate conversation on the appropriate method of reimbursement for AI systems. Abramoff et al. highlight a per-use reimbursement framework that incorporates access to care concerns and discuss the relevance of this model to the first CPT code for AI-performed diabetic retinal exams (IDx-DR, Digital Diagnostics Inc, Coralville, IA)^4^. Similarly, a recent commentary by Parikh and Helmchen reviews current models of payment for medical AI and explores five alternative and complementary reimbursement strategies for AI system reimbursement^5^. We discuss these two perspectives below.

## Per-use reimbursement and “access-maximizing value”

While fee-for-service models often set fees based on an estimate of value delivered, Abramoff et al. propose an additional consideration: access. Under this “access-maximizing” model, Medicare would incorporate a fixed adjustment for the proportion of eligible patients who receive the service. For example, if a procedure generates $100 of value but only half of the eligible patients receive it, Medicare may reimburse the procedure at $50 per patient. When applied to the IDx-DR model for diabetic retinopathy exams, we start with the value of an eye exam ($175, the median Medicare reimbursement) and multiply by the proportion of patients receiving eye exams as indicated (30%), arriving at $55. The authors observe that this $55 figure is consistent with the $45-64 (in relative value units) per exam assigned to IDx-DR in 2022. In this way, the authors argue that an “access-maximizing” method offers a useful starting point to set payment rates that can be easily integrated with current Medicare payment models like Physician Fee Schedule (PFS) and Resource Based Relative Value Scale (RBRVS).

This formula rewards efforts to improve the utilization of clinically beneficial services. However, AI manufacturers already have significant incentives to promote uptake, which may translate to increased sales. The formula may also excessively penalize AI services targeted at conditions that are underdiagnosed or underutilized due to factors beyond the control of AI manufacturers. Additionally, the difference between the reimbursed value of an eye exam and of IDx-DR may be attributable to several factors, such as an eye exam’s ability to diagnose conditions other than diabetic retinopathy. Moving from fee-for-service toward value-based reimbursement may better account for situations where functions of AI systems cannot be appropriately itemized into discrete services while also continuing to incentivize efforts toward expanding patient access.

## Alternative and complementary reimbursement approaches

Parikh and Helmchen discuss the unique drawbacks of fee-for-service models (e.g. per-use methods suggested by Abramoff et al.) for AI, including the near-zero marginal costs, risk, and ease of overutilization, and non-discrete value generated by some AI system services. The authors suggest a variety of reimbursement strategies that leverage private and public payers and providers, including less traditional approaches to incentivizing and guiding AI innovation and adoption. Importantly, these options are not mutually exclusive but rather can be used in conjunction with any single reimbursement policy to incentivize the optimal R&D and utilization.

### No reimbursement of AI systems

Under this model, health systems would directly pay for AI as a cost of doing business. This would only be financially feasible if the AI system reduces costs or increases revenue, e.g., by increasing procedural volume. As with other subscription services, health systems could negotiate directly with manufacturers for volume-based pricing, including discounts, rebates, or profit sharing^5^. While some AI systems may be highly profitable under such a model and therefore may not rely on insurance reimbursement for adoption, this model may overemphasize diagnoses of low-value, high-revenue hospital services. Moreover, it forfeits a key post-approval lever for facilitating patient-centered and value-based adoption.

### Incentivize outcomes instead of volume

Payers could incentivize the use of AI that improves patient outcomes using validated quality metrics^5^. Value-based payment has gained increasing momentum over recent years, as exemplified by the Merit-based Incentive Payment System (MIPS) and Alternative Payment Models established by the Medicare Children’s Health Insurance Program and Reauthorization Act (CHIP) of 2015^2^. As these programs continue to gain ground, measurement and reporting of outcomes meaningful to patients^6,7^ may enable more judicious deployment of AI systems. The difficulty of defining value-based outcomes also presents challenges for this approach. As Goodhart’s law states, measures that become goals can often be “gamed” until they cease to become useful measures, with the potential for serious negative consequences. For example, CMS previously used pain scores to determine hospital payments, which it ceased based on growing concerns that these metrics incentivized over-prescription of opioids^8^. Nevertheless, we believe that quality metrics, when carefully designed and implemented, can provide a powerful means to align financial incentives with patient benefit.

### Advance market commitments

Another model involves payments by payers and regulators for responding to predetermined health care challenges^5^, such as with COVID-19 epidemiologic predictive models^9^. AI developers could be rewarded with direct prize payments, pre-bargained purchase agreements, or capital investment toward further technology development. Kaggle^10^ or XPrize^11^ style models, involving massive open competitions with defined criteria for success, are promising for projects that build on publicly available data without sizable capital requirements. Ultimately, these models may be most useful for proof-of-concept demonstrations, considering the significant effort and expenditures required to produce and validate clinical-grade applications.

### Time-limited add-on reimbursements

New AI systems could be reimbursed for a time-limited trial period when they are not sufficiently covered by existing payment systems. These payments, such as NTAP, are based primarily on costs incurred rather than value delivered^12^, and therefore may be less suitable for AI tools with near-zero marginal cost. Still, such measures provide a dynamic “bridging period” that may be appropriate for AI systems undergoing post-marketing surveillance, ultimately facilitating faster adoption and diffusion of AI innovation. After the trial period elapses, the associated costs may be re-evaluated and then appended to bundled or episode-based payments^13^. Such transitional payments are important to help bridge the chasm between approval and adoption. Widespread use of such transitional payments would increase the agility of health care innovation by facilitating the interaction of providers and innovators while also expediting the post-approval real-world testing process through trial implementations.

### Reward generalizability and bias mitigation

Generalizability refers to the external validity of the AI tool across populations, which primarily depends on the representativeness of its training data. Unfortunately, AI tools trained on homogenous populations or single sites may introduce bias for underrepresented populations^14,15^. Given the limitations of FDA evaluation^1^, financial determinations contingent on generalizable performance and bias mitigation may be valuable. Requirements may include detailed demographic reporting and adequate representation of protected groups.

The proliferation of AI presents sizable challenges for identifying and prioritizing innovations that offer substantive and cost-effective improvements to patient care. Reimbursement by payers currently stands as one of the final checkpoints for hundreds of FDA-approved AI systems awaiting broad clinical deployment. This positioning offers a clear opportunity to guide the development and adoption of innovations to improve clinical workflows, access to care, and ultimately, patient outcomes. Reimbursement models integrable into current payment schemes, including per-patient rates, have demonstrated feasibility for early AI products. In the future, alternative and complementary models like direct payment, value-based payments, targeted competition rewards, transitional add-on payments, and performance standards for reimbursement all present principled options for value-based AI. A successful reimbursement strategy will align interests across stakeholders toward the common pursuit of better, faster, and more affordable care.
